# High perirenal fat thickness predicts a greater risk of recurrence in Chinese patients with unilateral nephrolithiasis

**DOI:** 10.1080/0886022X.2022.2158870

**Published:** 2023-01-13

**Authors:** Haichao Huang, Shi Chen, Wenzhao Zhang, Tao Wang, Peide Bai, Jinchun Xing, Huiqiang Wang, Bin Chen

**Affiliations:** a Department of Urology, The First Affiliated Hospital of Xiamen University, School of Medicine, Xiamen University, Xiamen, China; b Department of Radiology, The First Affiliated Hospital of Xiamen University, School of Medicine, Xiamen University, Xiamen, China; c The Third Clinical Medical College, Fujian Medical University, Fuzhou, China

**Keywords:** Nephrolithiasis, recurrence, perirenal fat thickness, obesity

## Abstract

**Introduction:**

The aim of this study was to evaluate the association between recurrence-free survival (RFS) and perirenal fat thickness (PFT) in a cohort of Chinese population with unilateral nephrolithiasis.

**Methods:**

We retrospectively reviewed the medical records of 81 patients with unilateral nephrolithiasis in our center from January 2019 to June 2019. PFT measured on computed tomography (CT) scans was evaluated. Kaplan-Meier curves and log-rank tests were used to assess significant differences in RSF between high-PFT and low-PFT groups within sexes. Univariable and multivariable Cox regression analyses were used to evaluate the potential risk factors for renal stone recurrence.

**Results:**

High PFT was significantly associated with high BMI and hyperlipidemia (*p* = .003 and.047, respectively). The PFT of stone-bearing kidney was significantly greater than PFT of non-stone-bearing kidney (0.77 ± 0.60 cm vs. 0.67 ± 0.58 cm, *p* = .002) . During the follow-up periods (median 31 months), 21 (25.9%) patients experienced ipsilateral renal stone recurrence. In addition, Kaplan–Meier survival curves showed that patients with low PFT had a significant better RFS than those with high PFT (*p* = .012). In the univariable Cox analyses, male sex and high PFT were significantly associated with a poor RFS (*p* = .042 and .018, respectively). Moreover, both male sex and high PFT retained significance in the multivariable analyses (*p* = .045 and .020, respectively).

**Conclusions:**

Our findings suggested that PFT is a noninvasive and feasible parameter, which may help in the risk stratification of renal stone recurrence in the follow-up periods.

## Introduction

Nephrolithiasis remains a multifactorial disease. Associations between nephrolithiasis and metabolic abnormalities has been previously reported [1]. Given the parallel growth with the prevalence of nephrolithiasis [2], obesity has recently been speculated as a potential risk factor of stone formation. Most studies that link nephrolithiasis and obesity have examined in Western population and used body mass index (BMI, kg/m^2^) as a surrogate marker [3]. However, controversial results have been reported in Chinese population, whose lifestyles and dietary habitues were to a great extent different from those of Western population [4].

BMI is a self-reported parameter and only presents the overall obesity. It could not distinguish the muscle from fat content [5]. Perirenal fat, as a metabolically active endocrine organ, has been proposed as more accurate predictors of obesity-related morbidities and metabolic abnormalities [6–9]. Thus, we hypothesized that perirenal fat accumulation may be a potential risk factor of renal stone formation and recurrence. Recently, a study of Lama et al. with a small patient cohort (*n* = 40) showed that stone-bearing kidney has a greater perirenal fat volume (PFV) than non-stone-bearing kidney [10]. However, the calculation of PFV on computerized tomography (CT) scans requires the use of specific imaging software, which may be unavailable in many institutions. Perirenal fat thickness (PFT), which has been reported to be well-correlated with PFV and visceral obesity, is a simple and readily parameter and can be measured by both CT-scans and ultrasound [11]. In our study, we evaluated the difference of PFT measured by CT-scans between stone-bearing kidney and non-stone-bearing kidney, as well as the association between sex-specific PFT and the risk of renal stone recurrence in a relatively larger patient cohort (*n* = 81) from the southeast coast of China. We hope to describe the role of PFT in the risk stratification of renal stone recurrence.

## Methods

### Patients and data collection

A total of 81 patients with unilateral renal stone disease (RSD), who were treated with percutaneous nephrolithotomy (PCNL) or retrograde intrarenal surgery (RIRS) in our center from January 2019 to June 2019 were retrospectively recruited. This study was conducted with the approval from the institutional ethics committee of the First Affiliated Hospital of Xiamen University. Patient data and metabolic risk factors were collected from the medical records, including gender, age at initial diagnosis (50 years old or younger vs. older than 50 years old), BMI (self-reported weight/height^2^, less than 25 kg/m^2^ vs 25 kg/m^2^ or greater), history of diabetes mellitus (DM), hypertension and hyperlipidemia (HLD). Stone data were also collected, including stone diameter and chemical composition (calcium oxalate (CO) vs. non-CO) and history of nephrolithiasis. Age at the first-time presentation has been reported as a risk factor of stone recurrence [12]. While no definitive cutoff value of age was defined, the median value of age was used to distinguish young from old patients in the survival analyses. Patients who had no available preoperative CT scans (*N* = 0), insufficient follow-up period (less than 6 months after being stone free, *N* = 9) or residual stone burden (*N* = 3) were excluded. In addition, patients (*N* = 5) were excluded if they had a history of ipsilateral renal malignant tumor, hyperparathyroidism, impaired renal function (serum creatinine more than 1.5 mg/dl), infection stone, malformation of the urinary system, and other diseases that might affect the risk of renal stone recurrence along with patients less than 20 years old. According to the postoperative chemical composition of the stones, all the patients will receive recommendation on lifestyle changes, including increased fluid intake and diet alterations. Stone recurrence was defined as the radiographic findings of newly ipsilateral renal stone. Time to stone recurrence was defined as the interval between being stone free and stone recurrence [13,14].

### The measurement of PFT

**Figure 1.**
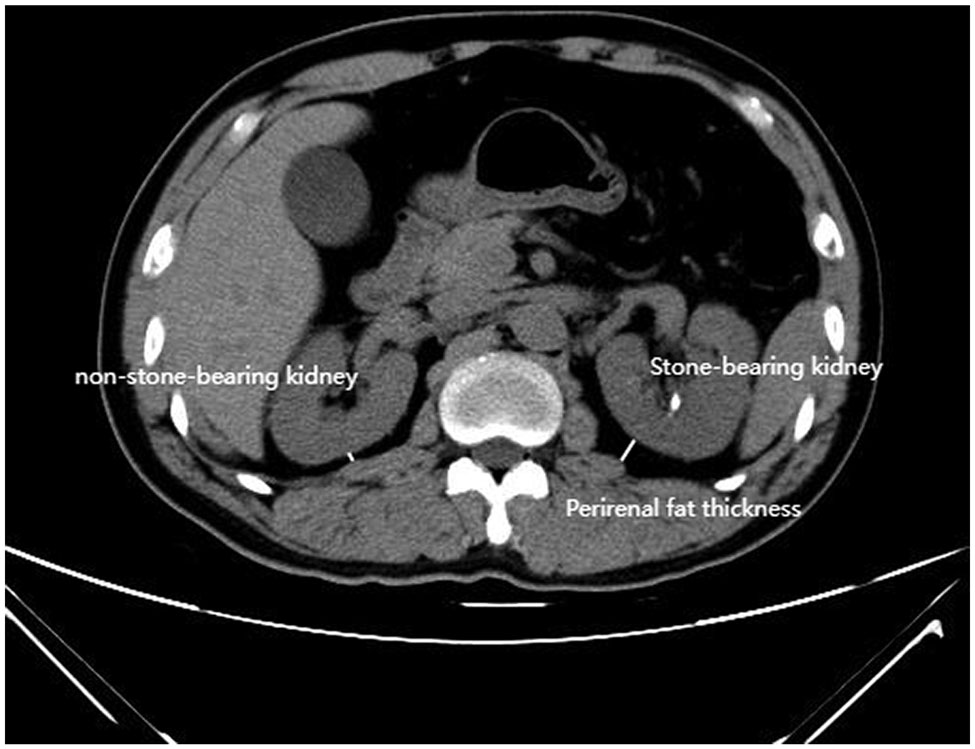
Axial CT scans at the level of renal hilum used for evaluating the perirenal fat thickness.

Routine abdominal CT scans (Somaris/7 CT 2012B, Siemens AG, Germany) were performed prior to the surgical intervention. All the CT scans were reviewed by a senior urologist (HCH) who was blinded to the clinical and survival data before the evaluation and under the supervision of a senior radiologist (SC). The Bland-Altman analysis showed an almost null mean difference between PFT of the stone-bearing kidney measured by SC and HCH (Supplemental Figure 1). The evaluation of PFT was in accordance with previous studies for both stone-bearing and non-stone-bearing kidney [15]. On routine CT scans, PFT (Figure 1) was defined as the shortest distance between the posterior surface of the kidney and the iliopsoas’s external margin at the level of renal hilum (renal vein) using the ruler function of the software. Given the difference in the value of stone-bearing kidney’s PFT between men and women, sex-specific median value [16] was used to dichotomize the entire cohort. As a result, patients were grouped as high-PFT (greater than 0.95 cm for males and 0.375 cm for females) and low-PFT groups during the survival analyses. Thus, 26 males and 12 females were classified as having high PFT values, whereas 29 males and 14 females were classified as having low PFT values.

### Statistical analysis

For statistical analysis, continuous variables were compared with t-tests, while categorical variables were compared with chi-square tests. Intra-group comparisons of PFT values between stone-bearing and non-stone-bearing kidney were performed using the paired t-tests. In the survival analyses, patients were dichotomized as high-PFT group and low-PFT group using sex-specific median value of stone-bearing kidney’s PFT. Kaplan–Meier survival curves were plotted and log-rank tests were used to evaluate the difference between the two groups. In addition, univariable and multivariable Cox analyses were performed to assessed the correlation between stone recurrence-free survival (RFS) and associated risk factors. All the statistical tests were performed using Statistic Package for the Social Science (SPSS) software (version 22.0), and statistical significance was defined as a *p* value of less than .05.

## Results

### Baseline characteristics

Fifty-five male patients and twenty-six female patients with a median age of 50 years old were enrolled in this study. High PFT, defined as greater than sex-specific median value, was significantly associated with high BMI and HLD (*p* = .003 and .047, respectively). There was no significant difference regarding age, history of DM, hypertension and nephrolithiasis, stone data (size and number) and surgical methods (PCNL vs. RIRS) between high-PFT group and low-PFT group. The baseline clinicopathological parameters of the two groups (low PFT vs. high PFT) were described in Table 1.

**Table 1.** Descriptive clinicopathologic characteristics of patients with unilateral nephrolithiasis (*n* = 81).

	Low-PFT group, n (%)	High-PFT group, n (%)	*p* Value
Age (year)			.338
≤ 50	26 (60.5)	18 (47.4)	
>50	17 (39.5)	20 (52.6)	
BMI (kg/m^2^)			.003
< 25	35 (81.4)	19 (50.0)	
≥ 25	8 (18.6)	19 (50.0)	
History of nephrolithiasis			.28
yes	11 (25.6)	6 (15.8)	
no	32 (74.4)	32 (84.2)	
Stone size (diameter, mm)			.596
< 10	5 (11.6)	2 (5.3)	
10 to 20	21 (48.8)	20 (52.7)	
≥ 20	17 (39.6)	16 (42.0)	
Stone number			.581
Single	7 (16.3)	8 (21.1)	
multiple	36 (83.7)	30 (78.9)	
Diabetes mellitus			.738
Yes	2 (4.7)	3 (7.9)	
No	41 (95.3)	35 (92.1)	
Hypertension			.399
Yes	6 (14.0)	8 (21.1)	
No	37 (86.0)	30 (78.9)	
Hyperlipidemia			.047
Yes	20 (46.5)	26 (68.4)	
No	23 (53.5)	12 (31.6)	
Treatment			.17
RIRS	23 (53.5)	26 (68.4)	
PCNL	20 (46.5)	12 (31.6)	

*Note:* High PFT: greater than 0.95 cm for males and 0.375 cm for females; BMI: body mass index; PFT: perirenal fat thickness; RIRS: retrograde intrarenal surgery; PCNL: percutaneous nephrolithotomy.

In total, 63 patients had postoperative stone analyses. There were 60 patients with CO-dominant calculi, whereas only 3 patients had Uric acid (UA) calculi. Male patients had a significantly greater BMI than female patients. There were only 4 female patients (4/26, 15.4%) had a BMI greater than 25 kg/m^2^, while 23 male patients (23/55, 41.8%) had a BMI greater than 25 kg/m^2^ (*p* = .018). Moreover, male patients had a significant greater PFT of the stone-bearing kidney than female patients (0.90 ± 0.61 cm vs. 0.48 ± 0.45 cm, *p* = .001). Of interest, the PFT of stone-bearing kidney was significantly greater than PFT of non-stone-bearing kidney (0.77 ± 0.60 cm vs. 0.67 ± 0.58 cm, *p* = .002) .

### Survival outcomes

**Figure 2.**
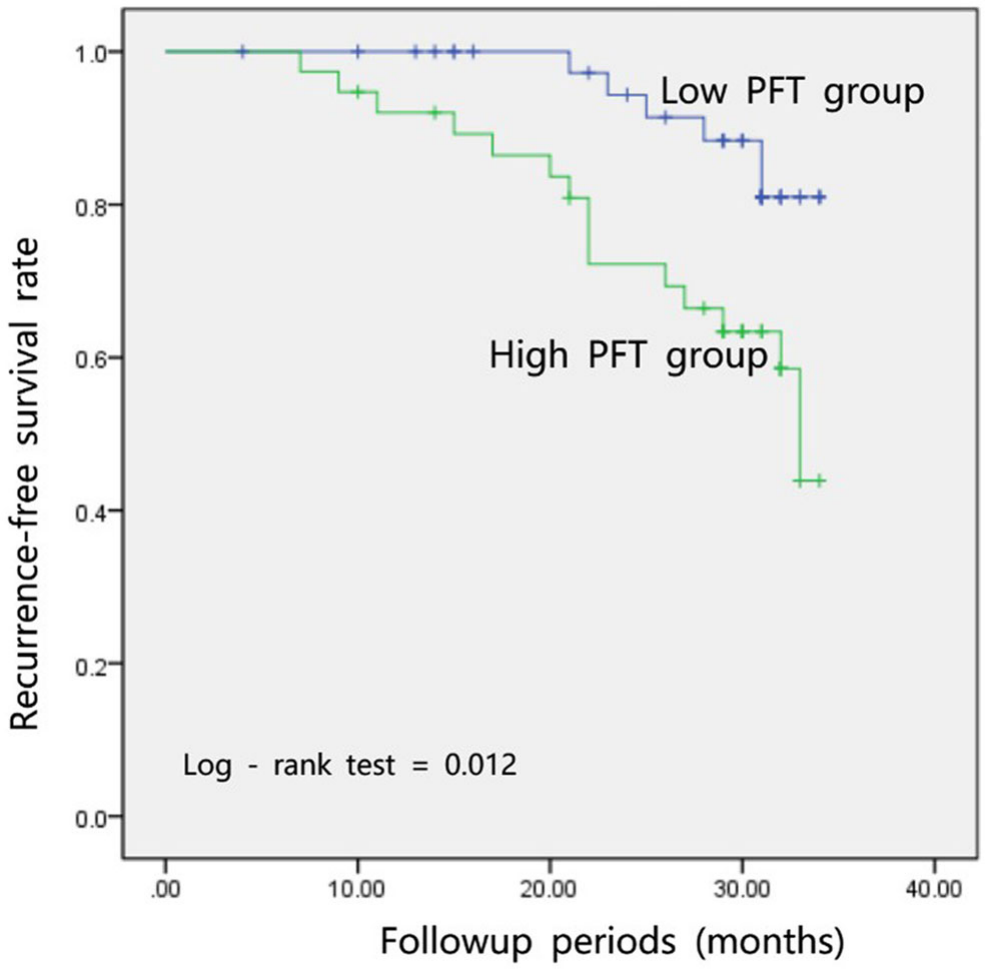
Patients with low perirenal fat thickness showed a significantly better recurrence-free survival than those with high value.

The median follow-up period was 31 months, with 21 (25.9%; 6 in low-PFT group and 15 in high-PFT group, respectively) patients experiencing ipsilateral renal stone recurrence during the follow-up period. Patients with low PFT showed a significant better RFS than those with high PFT (*p* = .012, Figure 2). In the univariable Cox analyses, high PFT and sex (male) were significantly associated with shorter RFS. Moreover, both high PFT and sex (male) retained a significant association with shorter RFS in multivariable Cox analyses (Table 2).

**Table 2.** Univariable and multivariable Cox regression analyses for prediction of recurrence-free survival in 81 patients with unilateral nephrolithiasis.

Univariable analyses	HR	95%CI	*p* Values
Age (> 50 y)	1.132	0.480–2.672	.777
Sex (male)	4.529	1.054–19.456	.042
BMI (≥ 25 kg/m^2^)	1.774	0.751–4.189	.191
PFT (high)	3.130	1.213–8.079	.018
Diabetes mellitus (yes)	0.635	0.084–4.776	.659
Hypertension (yes)	1.282	0.431–3.815	.655
Hyperlipidemia (yes)	0.596	0.235–1.405	.237
History of nephrolithiasis (yes)	0.382	0.089–1.643	.196
Stone size (large)	0.834	0.393–1.770	.637
Treatment (PCNL)	1.000	0.415–2.414	.999
stone number (multiple)	1.106	0.371–3.295	.856
Univariable analyses	HR	95%CI	*p* Values
Sex (male)	4.450	1.035–19.135	.045
PFT (high)	3.081	1.193–7.956	.020

*Note:* High PFT: greater than 0.95 cm for males and 0.375 cm for females; BMI: body mass index; PFT: perirenal fat thickness; CI: confidence interval; HR: hazard ratio.

## Discussion

Obesity, traditionally measured by BMI, has recently been speculated as a risk factor of renal stone formation [3]. Semins et al. reported that BMI greater than 30 kg/m^2^ was associated with a greater risk of RSD. In their study, those with a BMI greater than 30 kg/m^2^ had a higher incidence of renal stone than those with a BMI less than 30 kg/m^2^ (4.9% vs. 2.6%) [17]. However, the obesity rate of renal stone formers varied from different countries. Popov et al. evaluated the obesity rate (measured by BMI) of renal stone formers from different countries (*N* = 10). In their study, Chinese renal stone patients had the lowest obesity rate (2%), and the obesity rate in Chinese renal stone formers was not higher than the obesity rate in general Chinese population [18]. In a cross-sectional study of 10,281 Chinese rural participants, Fan et al. reported a similar level of BMI between patients with urinary stone disease (26.3 ± 3.8) and without urinary stone disease (26.3 ± 3.7) [4]. Moreover, the association between BMI and renal stone recurrence rate also remains controversial. In a prospective cohort study of 110 renal stone patients with a median follow-up period of 62 months, Bos et al. did not observe a significant difference between obese patients (defined as BMI ≥ 30 kg/m^2^) and non-obese patients in five-year stone-free rate (71.1% vs. 71.6%, respectively) [19]. In our study, BMI (25 or greater than 25 kg/m^2^) was not significantly associated with RFS of renal stone after a median follow-up period of 31 months (Table 2), which is consistent with the result of Bos et al.

Previous studies had reported a link between RSD and a number of health issues, including diabetes, hypertension, chronic kidney disease (CKD), and metabolic syndrome (MS) [20]. Moreover, perirenal fat tissue, as a metabolically active endocrine organ, has been reported to be associated with many types of aforementioned chronic diseases. In a cohort of 42 overweight and obese patients, De Pergola et al. demonstrated a positive independent association between perirenal ultrasonographic fat thickness and mean 24-h diastolic blood pressure levels [6]. Most recently, Ricci et al. observed a similar correlation between perirenal ultrasonographic fat thickness and hypertension in a larger cohort of morbidly obese patients [7]. Roever et al. in a cross-sectional study with 101 volunteers, reported that perirenal ultrasonographic fat thickness was significantly associated with the levels of fasting plasma glucose and metabolic syndrome in men, and with the levels of fasting plasma glucose in women [8]. Fang et al. reported a study aiming to evaluate the relationship between perirenal ultrasonographic fat thickness and estimated glomerular filtration rate (eGFR) in a cohort of 171 patients with type 2 diabetes, in which they observed that patients with higher PFT had lower eGFR [9]. Thus, we hypothesized that perirenal fat accumulation may be a better surrogate of obesity than BMI in predicting the risk of renal stone formation and recurrence.

Recently, a study by Lama et al. [10], in 40 patients with unilateral nephrolithiasis undergoing percutaneous nephrolithotomy as the primary treatment, showed that the mean PFV of stone-bearing kidneys was significantly greater than non-stone-bearing kidneys.

These findings were further substantiated by the study of Tastemur et al., where more participants were included [21]. However, without survival data in their studies, the association between perirenal fat accumulation and the risk of renal stone recurrence was unknown. Furthermore, although PFV can well reflect the entire perirenal fat accumulation, three-dimensional imaging software and other special programs, which may not be available in most of developing countries, are required. As PFT is not yet a well-established marker for renal stone formation and recurrence, using PFV as a surrogate of obesity may limit its potential use in further studies and daily clinical practice. Favre et al., in a cohort of 40 overweight and obese volunteers, reported that PFT measured by CT scans was well correlated with PFV and suggested PFT to be a simple and reliable estimate of PFV [11]. Furthermore, PFT can also be measured using ultrasound, which makes it more conveniently in the daily clinical practice. Thus, we hypothesized that PFT values may be associated with the risk of renal stone formation and recurrence. In our study, the PFT of stone-bearing kidney was significantly greater than PFT of non-stone-bearing kidney (0.77 ± 0.60 cm vs. 0.67 ± 0.58 cm, *p* = .002), which showed the important role of PFT in the formation of renal stone. Moreover, high sex-specific PFT of the stone-bearing kidney was significantly associated with poor RFS in both unilateral and multilateral analyses, which added survival data presenting the role of PFT in risk stratification of the recurrence of renal stone. In our study, male sex was significantly associated with poor RFS of renal stone in both univariable and multivariable Cox analyses (*p* = .042 and .045, respectively), which is consistent with the result of previous studies [12]. Of interest, male patients had a significantly greater PFT of the stone-bearing kidney than female patients (0.90 ± 0.61 cm vs. 0.48 ± 0.45 cm, *p* = .001), which may explain the difference in RFS between male patients and female patients in our study.

Little is known about the biochemical mechanisms that explain the association between perirenal fat accumulation and the formation and recurrence of RSD. BMI is related with changes in the biochemical components of urine, as well as urinary pH [2]. In a recent study of Trinchieri et al. [22], overweight and obese patients showed a significantly higher level of urinary excretion of calcium and oxalate, which are risk factors for stone formation. With the fact that the main component of kidney stones in our current study was calcium oxalate, we suggested that these possible associations may also exist between PFT and RSD. In the study of Lama et al. [10], PFV of stone-bearing kidney was significantly higher than the PFV of non-stone-bearing kidney in patients specially with CO-dominant stones. They hypothesized that the CO-dominant stone is a pan-renal inflammatory condition that may impact the PFV. However, we did not have sufficient data relating to the biochemical components of urine in this study, where further studies are certainly needed.

Dyslipidemia has also been reported to be associated with calcium kidney stone disease. In a recent study, Huang et al. demonstrated a significant link between dyslipidemia and calcium kidney stone disease, where a higher serum total cholesterol (TC) and low-density lipoprotein (LDL) were all associated with lower urinary citrate in calcium oxalate stone formers. They also reported a significant higher level of oxidative stress markers in the higher serum TC and LDL calcium-containing stone disease subgroups [23]. Tastemur et al. reported that the number of patients with HLD was significantly higher in kidney stone group than control group, and the presence of HLD was an independent risk factor for the presence of renal stones [21]. In the study of Lama, the number of patients with HLD was higher in CO-dominant stone subgroup [10]. In our study, although HLD did not present as an independent risk factor of stone recurrence in the Cox analyses, we did observe a significant link between PFT and HLD. Patients in the high-PFT group had a significantly higher risk of HLD than those in the low-PFT group. Moreover, recent experimental studies had described a link between perirenal fat and renal endothelial dysfunction, which has been reported to be a common etiology of chronic kidney disease, as well as a potential cause of urolithiasis [24,25]. In addition, CKD leads to the dysregulation of fibroblast growth factor 23 (FGF23)-Vitamin D (Vit. D)—parathyroid hormone (PTH) axis, which has been proven to be associated with higher risk of symptomatic kidney stones [26].

To the best of our knowledge, we are the first to evaluate the association between PFT and the formation and recurrence of RSD in a Chinese population, which were significantly different from American or European populations in the distribution of obesity. In our study, the PFT of the stone-bearing kidney was higher than non-stone-bearing kidney. Moreover, high PFT and male sex were all associated with a higher risk of stone recurrence. We suggested that PFT may be a readily available and significant predictor of renal stone formation and recurrence. Nonetheless, we did not evaluate the potential mechanisms underlying the association between PFT and the development of RSD in our study, which is a limitation of our study in addition to its retrospective nature. Furthermore, the number of patients with UA stones was markedly small (*n* = 3), while the majority of patients were CO-dominant stones in our study. Future studies with larger sample size of other stone types are needed to evaluate the association between PFT and stone types. In addition, the relatively small sample size and short follow-up period represent other limitations of our study.

## Conclusion

PFT is a significant predictor factor of the formation and recurrence of RSD in a Chinese population. We suggest that this noninvasive and feasible parameter may help in the risk stratification of renal stone recurrence during the follow-up periods.

## Supplementary Material

Supplemental Material
