# Supplementary figures and images for: High perirenal fat thickness predicts a greater risk of recurrence in Chinese patients with unilateral nephrolithiasis
Source: Ren Fail. 2023 Jan 13;45(1):2158870. doi: 10.1080/0886022X.2022.2158870 (PMC9848376; doi:10.1080/0886022X.2022.2158870)

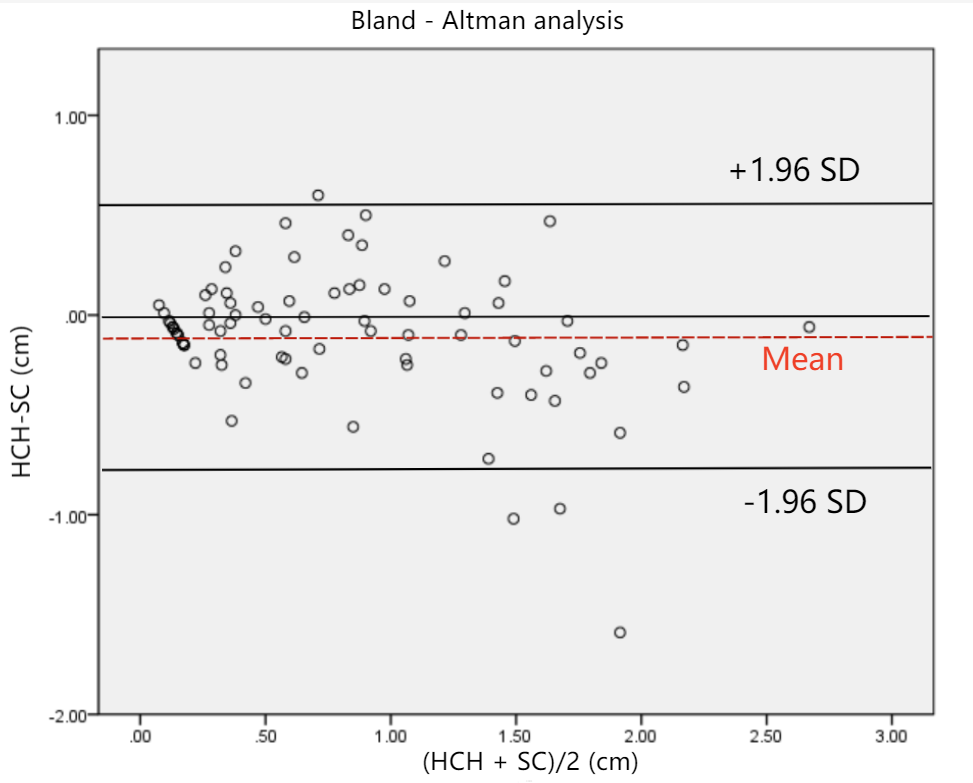

Supplement: Supplemental Material [file IRNF_A_2158870_SM6561.png]
